# Experiences, attitudes and barriers towards research amongst junior faculty of Pakistani medical universities

**DOI:** 10.1186/1472-6920-9-68

**Published:** 2009-11-16

**Authors:** Saniya Sabzwari, Samreen Kauser, Ali Khan Khuwaja

**Affiliations:** 1 Department of Family Medicine, Aga Khan University, Karachi, Pakistan; 2 Department of Community Health Sciences, Aga Khan University, Karachi, Pakistan

## Abstract

**Background:**

The developing world has had limited quality research and in Pakistan, research is still in its infancy. We conducted a study to assess the proportion of junior faculty involved in research to highlight their attitude towards research, and identify the factors associated with their research involvement.

**Methods:**

A cross-sectional study was conducted in four medical universities/teaching hospitals in Pakistan, representing private and public sectors. A pre-tested, self-administered questionnaire was used to collect information from 176 junior faculty members of studied universities/hospitals. Logistic regression analysis was used to identify factors related to attitudes and barriers in research among those currently involved in research with those who were not.

**Results:**

Overall, 41.5% of study subjects were currently involved in research. A highly significant factor associated with current research involvement was research training during the post-graduate period (p < 0.001). Other factors associated with current involvement in research were male gender, working in the public sector and previous involvement in research. Overall, a large majority (85.2%) of doctors considered research helpful in their profession and had a positive attitude towards research; nevertheless this positive attitude was more frequently reported by doctors who were currently involved in research compared to those who were not (OR = 4.69; 95% CI = 1.54-14.26). Similarly, a large proportion (83.5%) of doctors considered research difficult to conduct; higher by doctors who were not presently involved in research (OR = 2.74; 95% CI = 1.20-6.22)

**Conclusion:**

Less than half of the study participants were currently involved in research. Research output may improve if identified barriers are rectified. Further studies are recommended in this area.

## Background

Health research is essential in improving health care and plays a central role in the field of medicine. Advances in disease surveillance, diagnosis, treatment and prevention all rely heavily on quality research. In addition research influences health care policy [1]. Critical thinking skills of individuals are also greatly enhanced as a result of their involvement in research. Clinicians incorporate information from clinical research trials [2] into their practices, which improves patient management and disease outcome. Globalization of diseases and the increasing need of incorporation of evidence based medicine into practice have made quality demographic and clinical research even more imperative.

For a long time most of the developing world relied on research findings, interpretation and application from the western world. This did not always provide the solution to the problems of developing countries. Slow advances however, have been made in medical research in developing countries [3] and more funding, material and logistic support has been provided for conducting research. Nevertheless, the quality of research is affected by lack of expertise in research skills [4]. Problems are also seen in sharing and dissemination of results locally [5] and in incorporation of research findings in policy making [4]; either because a lack of understanding of research findings or its clinical implications by the health policy makers.

Literature shows that clinicians' interest and involvement in research has declined in recent years [6,7]. Several studies have looked at attitudes and interest in research among doctors working in various specialties and subspecialties. In the primary care field, most studies found time, financial constraints [7], busy clinical practices [8] and lack of interest [9] as major deterrents to clinicians' involvement in research. Other similar studies identified financial incentives and infrastructure support as key factors in promoting research [10]. Age and gender differences in research interest were also seen with younger physicians showing more inclination towards research [11] and a comparatively smaller involvement of female physicians [7]. Inadequate mentorship and lack of time have been other major barriers in research [12,13]. Bland and Ruffin [14] and Brocato and Mavis [15] identified accessible resources, appropriate rewards, time allocation, promotion and tenure as stimulators for research and scholarly productivity.

Pakistan is teeming with a multitude of medical, environmental and psychosocial issues which are just waiting to be explored. However, research here like most developing countries is in its infancy. This country faces obstacles in medical research which are similar to other developing countries. With a few exceptions, there is little quality research in Pakistan and a large majority of work is compromised due to flawed methodology and poor research training and background of researchers [5].

Some efforts are being made to improve training in research both at undergraduate and post graduate level yet research output still remains low [3]. Most of the research being produced is through required papers generated by postgraduate trainees [16]; which is a mandatory requirement for their training. It is therefore important to understand and highlight the attitudes and problems of health care providers in conducting quality research. This may help identify barriers and further encourage research by young professionals so that future research is more in number, better in quality and greater in impact. This study aimed to assess the proportion of junior faculty members of Pakistan's teaching medical institutes who are currently involved in research and to identify the factors associated with their involvement in research. In addition, we also assessed the attitudes and barriers towards research among these participants.

## Methods

A cross-sectional study was conducted in four medical universities/teaching hospitals in Pakistan: two each from the public and private sector. Self-administered and anonymous questionnaire was distributed to 220 eligible consenting study participants. The questionnaire was collected back after completion within one week of distribution. For those who had not returned the completed questionnaire by the due date, a reminder was given and another week was offered for completion of the questionnaire. Full confidentiality of the data collected was ensured to all the study participants and their representing universities/hospitals. They were also assured that the results of this study would not be presented either at an individual study participant or university/hospital level. Even though, no harm was expected to occur to any of the participating faculty and/or university, the study questionnaire was reviewed and approved by the Research Committee of the department of Family Medicine, Aga Khan University, Karachi. All data collection was done by medical graduates who received prior training for this task.

A structured questionnaire was designed incorporating important barriers and attitudes in research that were identified through an extensive literature search of the Pub Med database. After consensus of all study investigators, we also included some questions, which were particularly important to our local scenario. Questions about past research involvement and experiences were also included. The time required to complete the questionnaire was about 10 to 12 minutes. In all, 18 questions were included in the study questionnaire. The first five were about study participants' back ground information; the next seven about research training, publications, projects and grant obtained; followed by two questions each about research attitudes, barriers and future plans. The format of all the responses was in categorical design (yes/no and by choosing appropriate responses among already given options). Face and content validity of the questionnaire was obtained through a review process with experts in the field. After incorporating the identified inconsistencies and inaccuracies, the questionnaire was pre-tested on a group of family medicine residents to identify any problems relating to question design, flow and interpretation. This was also done to ensure that the questionnaire was in concordance with the study objectives. Suggestions given were incorporated accordingly.

This study included only those faculty members who were working as Junior faculty (Fellows, Instructors, Senior Instructors and Assistant Professors) at participating universities/hospitals. Senior faculty at associate professor and above were not included and interviewed in this study, as in our setting prior research done during the course of their careers and current seniority made their knowledge, experiences and needs different from junior faculty. We also excluded those faculty members who have reviewed the study questionnaire and protocol previously during its development and finalization phase. Faculty members from the departments of community health sciences and basic health sciences were also not included in this study, as their training and focus in research was more structured and comprehensive compared to other departments making them more productive in research.

Data was analyzed using Statistical Package for Social Sciences (SPSS) version 16. The proportion of junior faculty members who were currently involved in research was calculated. Chi-square test was used to assess the association between the outcome variable (current involvement in research) and the variables related to study participants' work and training. The differences in proportion of attitude and barriers towards research were calculated between those who were currently involved in research versus those who were not involved by logistic regression analysis with 95% confidence intervals of odds ratios.

## Results

In all, 220 eligible study participants were approached; out of which 191 responded (response rate was 87%). However, in final analysis we only included 176 completed questionnaires. Majority of the junior faculty members working in medical universities of Pakistan were men 35 years and older. Majority (56.2%) of the study subjects were working in medicine and allied specialties and 67.6% in private sector universities. Almost equal percentages of faculty were working at Level I (Fellows, Instructors and Lecturers) and Level II (Senior Instructors and Assistant Professors). A very small proportion (6.8%) of study participants received research training during their undergraduate years, while majority (63.1%) of the subjects had obtained research training during postgraduate studies. A large number (59.7%) of study participants had prior research involvement and about half of them had at least one research publication and presentation to their credit (table 1).

**Table 1:** Proportion and factors associated with present involvement in research among junior faculty members of teaching hospitals in Pakistan

Factors	Total faculty (n = 176) Percentages	Percent presently involved in research (n = 73)	Percent presently not involved in research (n = 103)	p-value
Sex				0.01
- Women	44.3	32.9	52.4	
- Men	55.7	67.1	47.6	

Age in years				NS
- < 35	46.6	49.3	44.7	
- ≥ 35	53.4	50.7	55.3	

Department				NS
- Surgery and allied	43.8	37.0	48.5	
- Medicine and allied	56.2	63.0	51.5	

Type of university				0.04
- Private sector	67.6	58.9	73.8	
- Public sector	32.4	41.2	26.2	

Designation				NS
- Level I*	51.1	50.7	51.5	
- Level II**	48.9	49.3	48.5	

Previous research training during undergraduate stage				NS
- No	93.2	90.4	95.1	
- Yes	6.8	9.6	4.9	

Previous research training during postgraduate stage				< 0.001
- No	36.9	16.5	51.5	
- Yes	63.1	83.6	48.5	

Previous research involvement				0.02
- No	40.3	30.1	47.6	
- Yes	59.7	69.9	52.4	

Previous research publication (s)				0.05
- No	51.1	42.5	57.3	
- Yes	48.9	57.5	42.7	

Previous research presentation (s)				0.05
- No	48.3	39.7	54.4	
- Yes	51.7	60.3	45.6	

* Level I: Fellows, Instructors and Lecturers

**Level II: Senior Instructors and Assistant Professors

NS: Not significant

In all, 41.5% of study participants were currently involved in research; a significantly higher proportion of men compared to women (men: 50.0% and women: 30.8%; p = 0.01) and those working in public sector versus private sector universities (public: 52.6% and private: 36.1%; p = 0.04). Faculty working in medicine and allied department(s) were currently involved in research at a higher proportion compared to their counterparts in surgery and allied department(s); however the difference was not statistically significant. The most significant factor associated with current involvement in research was research training during postgraduate education (p < 0.001). Those who had research training during their postgraduate years were involved in current research at a significantly higher proportion 55.0% compared to those who had not, 18.5%. Similarly those who were previously involved in research and had research publications and presentations were currently involved in research at significantly higher proportions (table 1).

Attitudes and barriers towards research and differences amongst those currently involved in research versus those who were not, are given in table 2. Overall, a preponderance of study subjects reported a positive attitude toward research particularly those who were currently involved in research. A large majority (85.2%) of study participants agreed that research was helpful and this proportion was significantly higher among those currently participating in research (94.5%) as compared to those who were not participating in research (78.6%) (OR = 4.69; 95% CI = 1.54-14.26; p = 0.003). Amongst doctors who reported that research as helpful; a higher proportion of those who were presently involved in research reported that research promote critical thinking as compared to those who were not involved in research (AOR = 2.48; 95% 1.22-5.05). Similarly, about half of the study subjects reported that research improve patient care and this positive attitude was higher among those who were involved in current research (AOR = 2.11; 1.05-4.25) (Table 2).

**Table 2:** Percentage distribution and factors associated with attitude and barriers towards research among junior faculty members of teaching hospitals in Pakistan

Statements agreed	Percent presently involved in research	Percent presently not involved in research	Odds Ratio (95% CI)	Adjusted Odds Ratio (95% CI)
**Attitude towards research**				

Research is helpful	n = 69	n = 81		

- Promotes critical thinking	72.6	43.7	3.42 (1.79-6.51)	2.48 (1.22-5.05)

- Improves patients' care	67.1	39.8	3.09 (1.65-5.78)	2.11 (1.05-4.25)

- Helps in promotion	61.6	44.7	1.99 (1.08-3.67)	

- Helps professional enhancement	68.5	54.4	1.83 (0.97-3.41)	

- Helps to changes health policy	50.7	48.5	1.03 (0.49-2.14)	

**Barriers towards research**				

Research is difficult	n = 55	n = 92		

- Lack of research allotted time	64.4	68.0	1.17 (0.62-2.21)	

- Lack for research training	52.1	68.0	1.95 (1.05-3.63)	1.95 (1.05-3.63)

- Lack of statistical support	47.2	51.5	0.33 (0.34-1.17)	

- Lack of mentorship	42.5	48.5	1.29 (0.69-2.34)	

- Lack of financial incentives	28.8	33.0	1.22 (0.64-2.33)	

Over 83% of study subjects admitted that research was difficult to conduct and this is higher among those who were not involved in research currently (89.3%) as compared to those who were involved (75.3%) (OR = 2.74; 95% CI = 1.20-6.22; p = 0.01). Lack of training as barrier towards research was reported by 68.0% of study subjects who were not involved in current research compared to 52.1% by those who were currently involved in research (OR = 1.95; 95% CI = 1.05-3.62). The other barriers were almost equally identified by both the groups. (Table 2).

## Discussion

To the best of our knowledge, this study is the first attempt in Pakistan that looked at characteristics of physicians and challenges in research faced by early to mid career level physicians working in different academic settings (both private and public sector universities). This study is also the first attempt to highlight differences in attitudes and barriers towards research in physicians active in research versus those who are not.

Overall, majority of physicians sampled, were not involved in research. However, the number of physicians currently involved in research was surprisingly comparable, if not better, to similar studies [7,9,17] conducted in more developed parts of the world.

As expected, the number of male physicians working at a postgraduate level was higher in our institutes compared to females. Among the female faculty, involvement in research was significantly lower compared to males. This finding is similar to some studies done in the US [6,7], one of which [6] cites low self-ability as a major barrier toward involvement in research. No distinct reason was identified in our study to explain this occurrence. However, this difference may be due to different cultural and social expectations and responsibilities faced by females in our setting in contrast to males: like household and marital responsibilities.

Historically staff training, infrastructure, and resources are considered stronger in the private as compared to public or government run hospitals in Pakistan. Thus it was a surprising finding in our study that a higher number of physicians from the public sector were currently involved in research compared doctors in to private universities. This may be explained by a recent influx of funds and support provided to our public sector universities through large grants by Higher Education Commission of Pakistan. The quality of research being done in both the public and private sectors however was not assessed in this study.

Another important finding of this study was an almost lack of undergraduate training in research provided to our medical students. A similar study from India reported that 91% of interns had no research experience in medical schools [18]. Exposing medical trainees at an earlier stage in their careers to the basics of medical research not only improves knowledge and attitudes toward research but also helps to improve their skills in searching and critically appraising medical literature, independent learning and writing [19,20]. This training may translate into an increased interest in research at a post-graduate level and generation of research not only as mandatory projects but also as an additional if not exclusive arm of their careers. Most of the research training of our participants was obtained at a postgraduate level, which in turn was one of the most important factors associated with current involvement in research. Physicians with prior research experience, publications and presentations were more likely to have ongoing research projects. This may be explained by an increased experience and know how in performing literatures searches, data collection, analyses and interpretation. Appropriate rewards like promotions and publications have also been found to be strong motivators and predictors for researcher productivity [10,14,15]. This may very well explain the greater involvement in active research of such participants, also a significant finding of this study.

This study was also able to identify differences in attitudes toward research among study participants very clearly. There were statistically significant differences between faculty actively involved in research, who had a more positive outlook towards research both at a patient care and personal professional level as compared to faculty not involved in ongoing projects.

Another main objective of our study was assessment of barriers of which, a lack of research training was only barrier to have statistically significant difference between those involved in research versus not. This barrier was also cited in another study done earlier in Pakistan [21]. Allocation of time is identified as one of the characteristics of research productivity among faculty at US medical schools [15]. Majority of study participants also pointed out lack of time as one of the barriers for not doing research. This barrier is also reported by other researchers as well [7,9,12]. Even though no statistically significant differences were found between the two groups of physicians involved and not involved in research, other barriers selected by both groups were considerable in number and fairly similar. Of note was that lack of financial incentives was the least selected by both groups.

One limitation of this study was that we did not look at the type of research done by the study participants. Even though not a study objective, information of the quantity and quality of research would have given more information of research output and reflected on adequacy of research. Secondly, a larger sample size would have allowed for a stronger analysis than the one performed in this study. Statistical validation of the data collection tool was done, nevertheless its face and content validity was well conducted. Despite these limitations, this study was able to capture a wider sample across four academic institutions allowing for a better representation than in previous local studies.

## Conclusion

Majority of the junior faculty of Pakistani medical universities who participated in this study were currently not involved in research and a very small proportion of them received any training during their undergraduate studies. The most significant factor associated with involvement in current research was research training during post-graduation education. It is encouraging to note that in this study a large majority of doctors considered research helpful for their profession and had positive attitudes towards research. However, at the same time, the preponderance of participating faculty considered it difficult to conduct research, with the most common barriers being lack of time, research training, statistical support and mentorship.

Thus, we recommend addressing the gaps and barriers identified by study participants with effective interventions. Gender differences in research involvement also needs attention. Teaching and training in research should be made compulsory during undergraduate as well as post-graduate studies. Giving some protected research time and participation in research methodology workshops and courses should be made readily available for faculty, with provision of statistical assistance. In addition, availability and support of supervisors and mentors should be assured. This work also paves way for further such studies at a larger scale that look at the quality of research training and output resulting from it.

## Competing interests

The authors declare that they have no competing interests.

## Authors' contributions

SS conceived and designed the study and prepared the manuscript. SK drafted and administered the questionnaires and managed the data. AKK analyzed and interpreted the data and provided intellectual feedback and supervision throughout the study. All authors read and approved the final manuscript.

## Pre-publication history

The pre-publication history for this paper can be accessed here:

http://www.biomedcentral.com/1472-6920/9/68/prepub
